# European and American chestnuts: An overview of the main threats and control efforts

**DOI:** 10.3389/fpls.2022.951844

**Published:** 2022-08-24

**Authors:** Patrícia Fernandes, Maria Belén Colavolpe, Susana Serrazina, Rita Lourenço Costa

**Affiliations:** ^1^Instituto Nacional de Investigação Agrária e Veterinária, I.P., Oeiras, Portugal; ^2^Green-It Bioresources for Sustainability, ITQB NOVA, Oeiras, Portugal; ^3^Department of Environmental Biology, State University of New York College of Environmental Science and Forestry, Syracuse, NY, United States; ^4^BioISI – Biosystems and Integrative Sciences Institute, Faculdade de Ciências da Universidade de Lisboa, Lisbon, Portugal; ^5^Centro de Estudos Florestais, Instituto Superior de Agronomia, Universidade de Lisboa, Lisbon, Portugal

**Keywords:** breeding, *Castanea*, climate change, chestnut blight, *Cryphonectria parasitica*, drought, ink disease, *Phytophthora cinnamomi*

## Abstract

Chestnuts are multipurpose trees significant for the economy and wildlife. These trees are currently found around the globe, demonstrating their genetic adaptation to different environmental conditions. Several biotic and abiotic stresses have challenged these species, contributing to the decline of European chestnut production and the functional extinction of the American chestnut. Several efforts started over the last century to understand the cellular, molecular, and genetic interactions behind all chestnut biotic and abiotic interactions. Most efforts have been toward breeding for the primary diseases, chestnut blight and ink disease caused by the pathogens, *Cryphonectria parasitica* and *Phytophthora cinnamomi*, respectively. In Europe and North America, researchers have been using the Asian chestnut species, which co-evolved with the pathogens, to introgress resistance genes into the susceptible species. Breeding woody trees has several limitations which can be mostly related to the long life cycles of these species and the big genome landscapes. Consequently, it takes decades to improve traits of interest, such as resistance to pathogens. Currently, the availability of genome sequences and next-generation sequencing techniques may provide new tools to help overcome most of the problems tree breeding is still facing. This review summarizes European and American chestnut’s main biotic stresses and discusses breeding and biotechnological efforts developed over the last decades, having ink disease and chestnut blight as the main focus. Climate change is a rising concern, and in this context, the adaptation of chestnuts to adverse environmental conditions is of extreme importance for chestnut production. Therefore, we also discuss the abiotic challenges on European chestnuts, where the response to abiotic stress at the genetic and molecular level has been explored.

## Introduction

The genus *Castanea* belongs to the Fagaceae family, and it is constituted of three sections: *Eucastanon* (chestnuts), *Balanocastanon* (chinquapins), and *Hypocastanon* (the Henry chestnut). The most representative species and of greater economic importance are included in *Eucastanon*: European chestnut (*Castanea sativa* Mill.), the American chestnut [*Castanea dentata* (Marshall) Borkh.], the Chinese chestnut (*Castanea mollissima* Blume), and the Japanese chestnut (*Castanea crenata* Sieb. and Zucc.) (Vieitez and Merkle, 2005; Mellano et al., 2012). Recently, researchers may have discovered a new chestnut species. *Castanea alabamensis*, was considered a hybrid between *Castanea dentata* and *Castanea pumila* Mill. (Allegheny chinquapin), but it was identified as a distinct genetic and morphological group in North America (Perkins et al., 2021).

Chestnuts originated in eastern Asia (Japan and China), from where they dispersed and diverge through Europe and North America (Lang et al., 2007). Nowadays, they are found across the northern hemisphere. *Castanea sativa* is distributed in temperate and Mediterranean regions of Europe and Western Asia; *Castanea dentata*’s natural range is across the Appalachian Mountain region; *Castanea mollissima* is native to China; and *Castanea crenata* is distributed in the Korean Peninsula, Japan, and the temperate region of East Asia (Pereira-Lorenzo et al., 2012).

In Asia and Europe, chestnuts have been cultivated for decades and have great economical value for their nutritious nuts and quality timber. In North America, they were treasured for being a multipurpose key-stone forest tree very important for populations and wildlife in its natural range (Anagnostakis, 1987). In 2020, the total chestnut plantation area in the world was approximately 582,545 ha, and more than half of this area belongs to China. This translated into an annual world production of approximately 2,300,000 tons, where China is the leading producer with almost 1,750,000 tons, followed by Spain with 189,000 tons (Food and Agriculture Organization of the United Nations, 2022).

The American and European chestnuts are the most susceptible species to several stresses, mainly biotic. The once dominant American chestnut was decimated in the 20th century (Anagnostakis, 1987), while in Europe *C. sativa* was chastised and nut production declined 251,549 tons from 1961 to 2015 (Food and Agriculture Organization of the United Nations, 2022). Biotic and abiotic stresses in forest systems have not been as well studied at a genomic level as it has for herbaceous crops. The recent technological advances in molecular biology and next-generation sequencing (NGS) technologies (Grabherr et al., 2011) helped overcome several difficulties inherent to studying woody species such as chestnuts. The transcriptome is modifiable under different conditions, making it a great tool to explore stress response. Association mapping and genome-wide association studies (GWASs) allow the association between molecular markers and phenotype in complex populations (Badenes et al., 2016), which can help understand the genetic architecture of stresses. The genomic resources gathered in the last decades have increased our knowledge about *Castanea* spp. genetic diversity, phenology, adaptation, and interaction with biotic and abiotic stresses. Transcriptomes during stress response (Barakat et al., 2009, 2012; Santamaría et al., 2011; Serrazina et al., 2015), development of molecular markers (Martin et al., 2010; Pereira-lorenzo et al., 2010, Pereira-Lorenzo et al., 2011; Nishio et al., 2011; Kubisiak et al., 2013; Santos et al., 2015b), mapping and identification of Quantitative Trait Loci (QTL) (Kubisiak et al., 1997; Casasoli et al., 2004, 2006; Zhebentyayeva et al., 2014, 2019; Santos et al., 2017b) have set us a step closer to using genomic selection in breeding programs. Biotechnologies implemented in chestnuts also include large-scale micropropagation of improved genotypes and genetic transformation. Several *in vitro* culture techniques such as axillary shoot propagation, organogenesis, and somatic embryogenesis have been reported over the last decades (reviewed in Corredoira et al., 2017) and recent advances are still being published (Fernandes et al., 2020b; Liu et al., 2022). Genetic transformation can be a powerful tool to study the function of a gene or for crop improvement and has been an important milestone in chestnut research (Powell et al., 2019; Pavese et al., 2021a).

**FIGURE 1:**
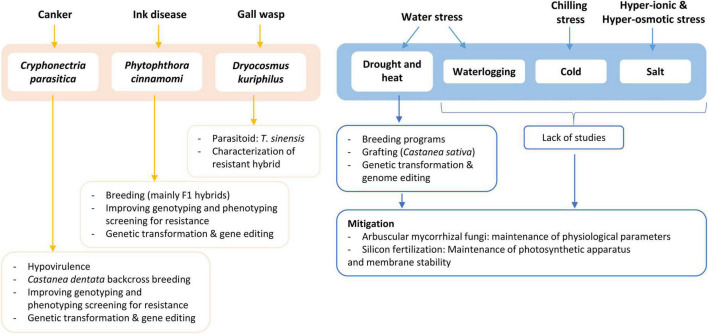
Schematic overview of the main topics discussed in this review. Information includes disease/stress, causal agent, and efforts for control/mitigation, in process or in perspective.

The research and breeding efforts made in the last decades seem to be having a positive impact since, in Europe, chestnut production has been increasing since 2015 for the first time in many decades (Food and Agriculture Organization of the United Nations, 2022). The purpose of this review is to provide a synopsis of the understanding gathered so far about chestnuts’ main biotic and abiotic challenges. The section on biotic stresses will have ink disease and chestnut blight as the main focus, while the abiotic section will mainly focus on Southern Europe, where there is a majority of reports. The development and application of biotechnologies are also discussed as they relate to the efforts of fighting European chestnut’s decline and revive the American species. Figure 1 briefly summarizes the main topics discussed in this review.

## Biotic stresses

*Castanea* species are challenged by several biotic stresses but the most destructive are *Cryphonectria parasitica* (Murr.) Barr. (*CP*) and *Phytophthora cinnamomi* Rands (*PC*), the pathogens causing chestnut blight and ink disease (also known as chestnut canker and root rot, respectively). Leaf spot (*Marssonina ochroleuca*), twig canker (*Cryptodiaporthe castanea*), and chestnut mosaic virus (ChMV) also affect chestnuts, however, the damage they cause is not as severe as the previously mentioned diseases (Serdar et al., 2019).

*CP* is a necrotrophic bark-inhabiting fungus whose primary hosts are *C. dentata, C. sativa, C. crenata*, and *C. mollissima*, although it also infects oaks (*Quercus* spp.), maples (*Acer* spp.), European hornbeam (*Carpinus betulus*) and chinquapins (*C. pumila* and *Castanea ozarkensis*). It is native to Eastern Asia, and it spread to North America and Europe due to imported infected chestnuts (Rigling and Prospero, 2018). *CP* was first described in 1904 in American chestnut in New York (Anagnostakis, 1987; Rigling and Prospero, 2018). In 50 years, it caused one of the most enormous economic and ecological devastations, leaving the American chestnut functionally extinct by killing an estimated 4 billion trees (Anagnostakis, 1987). This species now survives as stump sprouts (due to repeated blight infections) which are reservoirs of germplasm (Kubisiak and Roberds, 2006). *CP* was detected in Europe for the first time in 1938 in Italy, from where it rapidly spread to the rest of the continent to countries such as France, Switzerland, Portugal, Spain, and Turkey (Rigling and Prospero, 2018). It threatened the European chestnut stands, affecting production. Still, *C. sativa* recovered from the disease. This recovery was mainly related to the natural occurrence of mycoviruses in Europe that can infect this fungus and attenuate its virulence (hypovirulence, a viral disease that makes the pathogen less aggressive) (Robin and Heiniger, 2001; Milgroom and Cortesi, 2004). Also, *C. sativa* has lower susceptibility to the pathogen when compared to *C. dentata* (Waldboth and Oberhuber, 2009). Nowadays, *CP* is spread across Europe, North America, Africa (Tunisia), Asia and Australia (Eppo Global Database, 2022).

*PC* is a devastating hemibiotrophic pathogen with an extensive host range of close to 5,000 plant species (Hardham and Blackman, 2018). It has significant environmental and economic impacts (Weste and Marks, 1987; Hardham, 2005; Kamoun et al., 2015) by infecting plants important for forestry, and agriculture, such as chestnut, avocado, macadamia, oak, peach and pineapple [reviewed in Hardham and Blackman (2018)], causing annual damages of billions of dollars. *PC* is considered one of the Top 10 Oomycete plant pathogens (Kamoun et al., 2015). Its origin remains uncertain, but evidence indicates it originated near Papua New Guinea and South-East Asian regions (Ko et al., 1978; Zentmyer, 1988; Hardham, 2005). Ink disease was observed in American chestnuts and chinquapins in the southern United States since about 1850 [Milburn and Gravatt, 1932 as cited in Anagnostakis (2012)] and in Portugal since 1853 [Prunet, 1904 as cited in Anagnostakis (2012)]. But the first reports on this disease being caused by *PC* were a few years later. In European chestnuts, it was in 1860 [Grent, 1961 as cited in Burgess et al. (2017)], and in American chestnuts in 1986 [Corsa, 1986 as cited in Anagnostakis (2001)]. *PC* was introduced in all continents by plant-moving, except for Antarctica, becoming invasive worldwide (Eppo Global Database, 2022).

*Castanea* species have different susceptibility levels to these pathogens. The Asian species are the most resistant, probably due to their co-evolution with the pathogens (Crandall et al., 1945; Huang et al., 1996). In the specific case of chestnut blight, varying levels of quantitative resistance have been reported for Asian species, however, Chinese chestnut is considered more blight-resistant than Japanese chestnut (Huang et al., 1996).

Breeding for pathogen resistance in Europe and North America has different goals. The first focus on development and preservation of cultivars and ink disease-resistant rootstocks to reduce mortality, improve orchard production, and avoid further decline of the species; the latter seeks to restore the American chestnut as a forest species. What is common in these efforts is the use of the Asian species resistance and the high interspecies crossability, to introgress resistance genes into the susceptible European and American species (Burnham et al., 1986; Fernández-López et al., 2001; Pereira-lorenzo et al., 2010; Costa et al., 2011; Steiner et al., 2017). In Europe, blight infection has been under control due to hypovirulence and consequently, most research is focused on understanding the interaction with *PC*, mainly in *C. sativa* and *C. crenata.* Contrary to North America, where dedication goes to *C. dentata* and *C. mollissima* responses to *CP*. Nevertheless, in recent years North Americans realized that *PC* will be a problem to the cultivation of the American chestnut with improved blight resistance, mainly in the south where temperatures allow ink disease establishment (Jeffers et al., 2009).

During the last decades, chestnut breeding programs have been looking into *Castanea* spp. cellular and molecular responses to *CP* and *PC*, which may be comparable since Oomycetes and Fungi share similar infection mechanisms (Latijnhouwers et al., 2003). Several hypotheses have been proposed so far and are discussed in the next sections.

Biotic stresses also include several pests such as the gall wasp [*Dryocosmus kuriphilus* (Yasumatsu)], chestnut tortrix moths (*Cydia splendana*, *Cydia fagiglandana*, and *Pammene fasciana*), the chestnut weevil (*Curculio elephas*) and ambrosia beetles [*Anisandrus* (*Xyleborus*) *dispar*]. Less damaging pests currently affecting chestnuts are the potato leafhopper (*Empoasca fabae*), Japanese beetle (*Popillia japonica*), Rose chafer (*Macrodactylus subspinosus*) and spider mites. Also, Peach moth (*Dichocrocis punctiferalis*) and goat moth (*Cossus cossus*) were reported as important pests in Japan and Turkey, respectively [reviewed in Serdar et al. (2019)]. The gall wasp is the most globally significant pest. It attacks European, American, and Asian chestnut species, and their hybrids, reducing the quality and quantity of timber (Kato and Hijii, 1997) and nuts (Battisti et al., 2014). The gall wasp does not kill the trees and for that, it has been given less attention than the previously referred pathogens. However, its spread may erase all the breeding efforts toward European and American chestnut sustainability due to the reduced nut production. Also, galls can be an entry point for *CP* increasing branch mortality (Meyer et al., 2015). The next sections provide an overview of our current knowledge of the chestnut’s interaction with these three biotic stresses, with a focus on the pathogens.

### *Castanea* spp. and *Cryphonectria parasitica*

#### Infection progress and symptoms

The histopathology of *CP* infection progress is described in American and Chinese chestnuts (Hebard et al., 1984). After spore germination, the fungus develops mycelial fans, which, by physical pressure, colonize the host cells progressing intercellularly through the bark and cambium. The extent and frequency of mycelial fan formation are essential for the enlargement of cankers. The host responses against the infection are lignification of cell walls succeeded by wound periderm formation. Lignification only blocks individual or small aggregates of hyphae, and only fully formed wound periderm can stop mycelial fans (Hebard et al., 1984). The advance of the mycelial fans kills the host cells by releasing toxins and cell wall-degrading enzymes (Roane et al., 1986) and when the host does not develop deep wound periderms allows the pathogen to keep obtaining nutrients from dying and dead chestnut cells (necrotrophy) (Hebard et al., 1984). Oxalate (oxalic acid) was considered linked with *CP* virulence when researchers suggested it had a toxic effect on host cells and enhanced cell wall degradation (Havir and Anagnostakis, 1983). This was later confirmed in knockout mutants of the pathogen (Chen et al., 2010).

**FIGURE 2:**
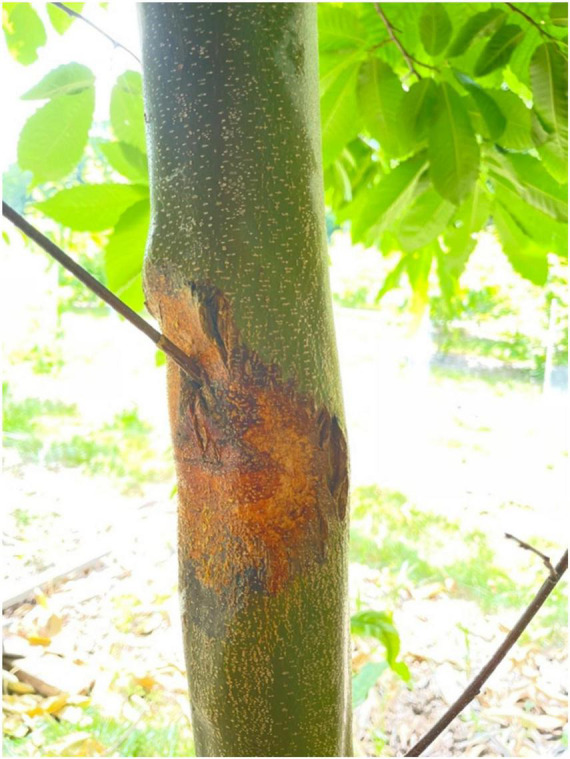
*Castanea dentata* infected with *Cryphonectria parasitica* presenting an orange canker on the main trunk. This picture was taken at the Lafayette Experimental Road Station – SUNY-ESF (Syracuse, NY, United States).

*CP* only infects above-ground tree parts, producing orange/reddish-brown cankers (necrotic lesions) on the bark (Figure 2) and killing smaller branches. An early symptom of infection is the wilted and hanging leaves on infected dead branches. Trees react by producing numerous epicormic shoots below the cankers (Rigling and Prospero, 2018). Blight-resistant chestnuts typically survive infection with minimal internal damage, developing superficial lesions or cankers on the trunk. In blight-susceptible species, disease symptoms usually progress rapidly, resulting in host mortality. Also, symptoms in susceptible hosts may vary depending on the virulence of the fungus, tree size, longevity, health, and environmental factors (Roane et al., 1986; Clark et al., 2019). *CP* can survive and sporulate on the bark of dead or recently dead chestnut branches or stems for more than 1 year (Prospero et al., 2006).

#### Chestnut blight control

Quarantine regulations were implemented worldwide to control the movement and trade of plant material infected with blight. Unfortunately, these measures were ineffective due to asymptomatic infected plants (Rigling and Prospero, 2018). In natural ecosystems, attempts to eradicate the pathogen or apply fungicides are not feasible. Cutting and burning infected trees is a management alternative, but only viable in orchards. The use of chemicals is restricted in forests because it can cause phytotoxicity, and may induce resistance.

Efforts to develop control methods for *CP* are summarized in Table 1. In Europe, the disease is successfully managed due to the sizeable natural occurrence of hypovirulence, a viral disease in *CP* population caused by double-stranded RNA viruses which reduces virulence and sporulation of strains. Contrary to North America where hypovirulence was only found outside the American chestnut range and there is limited success in viral transfer between different vegetatively incompatible fungus strains (Milgroom and Cortesi, 2004). Recent reports described a modification using genetically engineered “super donor” fungal strains that may help overcome these difficulties (Stauder et al., 2019).

**TABLE 1:** Summary of efforts to control *Cryphonectria parasitica* (*CP*).

Source of resistance/Plant material	Approach for improvement	Resources related to resistance		Current status/Outcomes	References
		Type of data	Description		
NA	Hypovirulence	NA	NA	Study of native hypovirulent strains of *CP*	Robin and Heiniger, 2001; Bryner et al., 2012; Murolo et al., 2018
NA	Hypovirulence: improve viral transfer in *CP*	NA	NA	Genetically engineered *CP* strain developed – “Super donor”	Stauder et al., 2019
*Castanea mollissima* varieties ‘Nanking’ and ‘Mahogany’	Backcross breeding of *Castanea dentata*	NA	NA	Selecting most resistant BC_3_F_2_ to intercross	Burnham et al., 1986; Diskin et al., 2006; Westbrook et al., 2020b
*C. mollissima* × *C. dentata* F2 hybrids	Study genetic architecture of *CP* resistance; future MAS	Genetic linkage map	3 QTLs: *Cbr1* (LG B), *Cbr2* (LG F), *Cbr3* (LG G)	NA	Kubisiak et al., 1997, 2013; Sisco et al., 2005
		Sequencing of *Cbr1, Cbr2*, *Cbr3*	Genes annotated with the term “defense response”. *Cbr1*: 8; *Cbr2*: 4; *Cbr3*: 3	NA	Staton et al., 2015
*C. dentata* and *C. mollissima*	Identification of resistance genes; future MAS	Transcriptome	Candidate genes related to: Cell wall biosynthesis, ROS, signaling of SA, ET, JA and ABA, HR, and PCD	NA	Barakat et al., 2009, 2012
*C. mollissima* × *C. dentata* BC_3_F_2_ generation and progeny (BC_3_F_3_)	Genomic prediction model for blight phenotypes	*C. dentata* draft genome; SNPs	GBS and phenotyping for canker severity on BC_3_F_2_, plus phenotyping BC_3_F_3_	Improving model: genotyping more BC_3_F_2_ trees	Westbrook et al., 2020b
*Oxalate oxidase* (*OxO*) gene from wheat	*C. dentata* genetic transformation	NA	NA	Tolerant American Chestnut waiting deregulation for restoration purposes	Powell et al., 2019; Newhouse et al., 2020

Source of resistance/plant material, approach for improvement, resources gathered, and current status and/or outcomes of these efforts are presented. NA, none applied; MAS, marker-assisted selection; QTL, quantitative trait loci; LG, linkage group; GBS, genotyping by sequencing; ROS, reactive oxygen species; SA, salicylic acid; ET, ethylene; JA, jasmonic acid; ABA, abscisic acid; HR, hypersensitive response; PCD, programmed cell death.

Due to the hypovirulence success in Europe most efforts to manage blight are focused on the study of hypovirulent strains of *CP* (Robin and Heiniger, 2001; Bryner et al., 2012; Murolo et al., 2018). North America is focused in developing a blight-resistant chestnut through traditional backcross breeding and genetic engineering. For this reason, the following sections on *CP* will be mainly focused on North American reports.

#### Traditional breeding for *Cryphonectria parasitica* resistance

American chestnut breeding for blight resistance has been ongoing for over 100 years. Inter-species crosses with Asian chestnut species (mainly Chinese) were started by the United States Department of Agriculture (USDA) and the Connecticut Agricultural Experiment Station (CAES) [reviewed in Jacobs et al. (2013)]. However, these programs failed to produce a blight-resistant tree that retained American chestnuts’ growth and quality timber. Besides the difficulties in finding the ideal candidate tree, nowadays we know there are other problems inherent to hybrid breeding that can turn restoration even more difficult such as male sterility (Sisco et al., 2014), internal kernel breakdown (IKB) (Fulbright et al., 2014) and intermediate traits (Cipollini et al., 2017).

In 1989 The American Chestnut Foundation (TACF) backcross breeding started at the Meadowview station by using two backcross hybrids (BC) [BC_1_ (*C. dentata* × *C. mollissima*) × *C. dentata*] from the USDA and CAES programs as two different sources of blight resistance: the ‘Clapper’ and ‘Graves’ (from *C. mollissima* variety ‘Nanking’ and ‘Mahogany,’ respectively). To achieve a population with Chinese chestnut resistance and the American chestnut phenotype, the original breeding plan (Burnham et al., 1986) proposed successive hybrid backcrossing with several pure American chestnut lines (to ensure genetic diversity), selecting for blight resistance and American phenotype in every progeny. This plan was based on the assumption that the alleles for resistance were partially dominant and only two genes were involved. Nowadays, we know that chestnut blight resistance is quantitative, involving three main resistance loci (of up to seven in total) (Kubisiak et al., 1997, 2013), which changed the backcross breeding stages to include three backcross generations. The third backcross would be intercrossed to produce a BC_3_F_2_ population in which a fraction of the trees was predicted to be homozygous for the Asian resistance alleles. The selected resistant individuals would be planted in seed orchards producing a BC_3_F_3_ generation that would be essentially American chestnut morphologically and blight-resistant enough to start restoration [reviewed in Jacobs et al. (2013)]. Indeed, the American phenotype was recovered in 96% of the BC_3_ generation, which resembled 24 measured traits (Diskin et al., 2006). Approximately 64,000 BC_3_F_2_ from both ‘Clapper’ and ‘Graves’ selections were planted between 2002 and 2018, and 7,600 trees remained as of 2018 (Westbrook et al., 2020b). Orchard trials of open-pollinated BC_3_F_3_ were made to estimate the genetic resistance of the selected BC_3_F_2_ trees, but after inoculations, the highest blight tolerance was more like American chestnut than Chinese chestnut. These findings suggested that blight resistance segregates at more loci than initially predicted and phenotypic selection has not been accurate enough (Steiner et al., 2017). This differs from 8-year-old BC_3_F_3_ forest reintroduction trials made in three locations in the southeastern United States, where the resistance of the trees was more like Chinese chestnut (Clark et al., 2019). TACF has ongoing field trials in 35 locations in the eastern United States but are still too young to assess blight resistance (Westbrook et al., 2020b). Nowadays, additional *C. mollissima* genotypes are being used as resistance sources (Steiner et al., 2017) at the Meadowview breeding station. This program has also been reproduced at 16 other locations by the different chapters of the foundation. After decades of breeding, the current goal is to select 1% of the 7,600 BC_3_F_2_ that are most blight-resistant, intercross the selected trees and increase BC_3_F_3_ blight resistance (Westbrook et al., 2020b). Also, selected blight-resistant hybrids are being evaluated for resistance to *PC* (Jeffers et al., 2009).

#### Screening for *Cryphonectria parasitica* resistant genotypes

Traditional screening for blight resistance can be determined by field inoculation of the stems/trunks (Griffin, 1983; Anagnostakis, 1992). These inoculations usually allow the evaluation of parameters such as mean canker length and width (canker expansion rate), stromata production, canker superficiality and swelling, and canker severity (Kubisiak et al., 1997; Steiner et al., 2017; Westbrook et al., 2020b). These methods are accurate but can only be performed in trees with at least 3 years of growth and cankers can take several months to develop. Alternative techniques can test younger plants (Powell et al., 2007; Newhouse et al., 2014). *CP* lesion progression can be accessed by small stem inoculations in trees with approximately 1-year-old and results can be collected in 3–4 weeks (Powell et al., 2007). However, this may harm the tree even if it has moderate levels of resistance. Alternatively, excised leaves from a few month-old seedlings can be inoculated (Newhouse et al., 2014). Leaves are not *CP*’s primary organ of infection. Nevertheless, results can be obtained in less than a week and correlate to stem inoculations (Newhouse et al., 2014), representing an expedited way to predict levels of blight resistance in big populations.

#### Improving characterization of *Cryphonectria parasitica* resistance

Kubisiak et al. (1997) developed a genetic linkage map with F_2_ hybrids from the backcross breeding program, mapping 184 molecular markers. In three different linkage groups (LG) seven QTLs related to blight resistance were reported. Three major QTLs explained about 40% of the phenotypic variation in canker size. This map was later expanded by Sisco et al. (2005) and Kubisiak et al. (2013) and the three major QTLs identified were sequenced (Table 1; Staton et al., 2015). Of 782 annotated genes, 15 were related to defense response, giving further insight about the candidate resistance genes (Staton et al., 2015). Barakat et al. (2009; 2012) also identified candidate genes for blight resistance by comparing American and Chinese chestnuts transcriptomes after *CP* challenge. The candidate genes were associated with response to biotic stimuli belonging to several pathways (Table 1; Barakat et al., 2012).

Westbrook et al. (2020b) recently suggested that blight resistance is a polygenic inherited trait. The population under study was the BC_3_F_2_ generation (mentioned in Section “Improving characterization of *Cryphonectria parasitica* resistance”). Genotyping-by-sequencing (GBS) and evaluation of different blight phenotypes in the BC_3_F_2_ population, along with canker severity assessment of their progeny (BC_3_F_3_), allowed the development of a genomic prediction model for blight resistance breeding (Table 1). They also performed GBS on *C. dentata* and *C. mollissima* to estimate hybrid indices.

### *Castanea* spp. and *Phytophthora cinnamomi*

#### Infection process and symptoms

**FIGURE 3:**
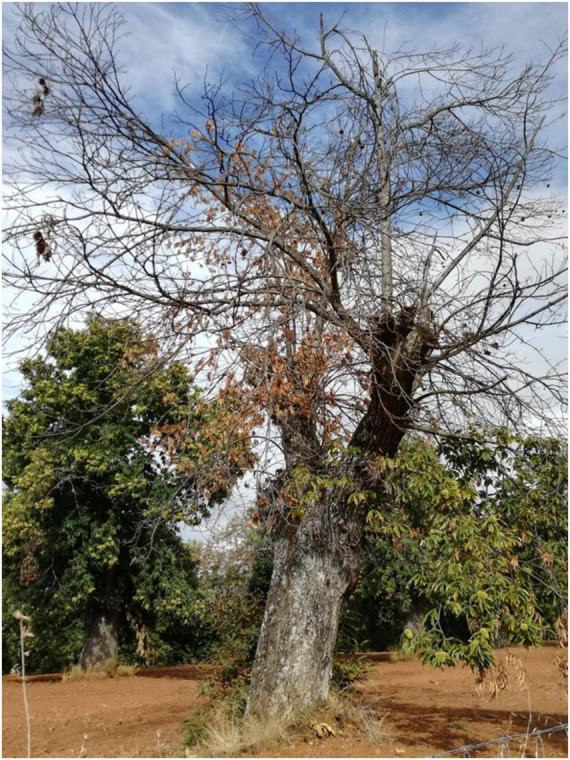
*Castanea sativa* shows symptoms of ink disease such as discoloration of leaves and dieback of branches. This picture was taken in Bragança, Trás-os-Montes region, Portugal.

The *PC* can saprophytically grow in the soil, and when conditions are favorable (high soil moisture and temperatures above 10^°^C) to sporulate it produces biflagellate motile zoospores (asexual spores) which seek out roots by chemotactically attracting to suitable infection sites (Carlile, 1983). The early stages of the infection process during *PC* infection have been characterized at the cellular level for *C. sativa* and *C. crenata* (Fernandes et al., 2021b). The zoospores shed their flagella, encyst, and grow a germ tube on the root surface until the development of an appressorium-like swelling that allows the rhizodermis penetration, initiating the infection process. The zoospores can identify and infect susceptible and resistant *Castanea* roots as quickly as 3.5 h after root inoculation. Hyphae develop until they reach the vascular tissues of the susceptible European chestnut on the third day of infection. At this stage, *PC* switches from biotrophy to necrotrophy, characterized by cellular collapse and it starts producing resistance structures (chlamydospores) in *C. sativa* (Fernandes et al., 2021b). Chlamydospores allow *PC* to persist in plant material and the soil for up to 6 years (Zentmyer and Mircetich, 1966). In the resistant chestnut, *C. crenata*, the infection progress is slower because the host can induce early defense responses, such as callose deposition, hypersensitive response-like, and production of phenolic-like compounds. Nevertheless, *PC* can still reach the vascular tissues (Fernandes et al., 2021b). After reaching the vascular tissues of susceptible chestnuts, the pathogen continues colonizing the roots until it obstructs xylem vessels (Gomes-Laranjo et al., 2004), preventing root growth and interfering with water and nutrient uptake to the shoot. The roots and root collar start to rot, resulting in a progressive decline of the tree (Hardham, 2005). The first above-ground symptom is the chlorosis and wilting of leaves at the top followed by the dieback of branches, defoliation, and gradual decline until the host dies (Figure 3; Gomes-Laranjo et al., 2004). In resistant chestnuts the progression of the lesion caused by *PC* seems to stop at the roots or root collar level (Santos et al., 2015a), preventing further decline of the tree.

#### Ink disease control

Control measures to prevent/restrain the pathogen have not been successful so far, mainly due to the easy development and migration of zoospores in humid conditions (especially during rainfalls) and to the resistance structures difficult to eradicate. Phosphite and metalaxyl have been the most efficient chemicals against *PC* [reviewed in Hardham (2005) and Hardham and Blackman (2018)]. However, the continuous use of these two chemicals has led to resistance development by the pathogen (Hardham and Blackman, 2018).

Until this date, there is no known effective biological control. Still, promising results were reported with soil inoculated with the bacteria *Byssochlamys nivea* or *Scopulariopsis brumptii*, which appears to decrease mortality in chestnuts inoculated with *PC* (Bosso et al., 2016). More common control approaches are the correct management of nurseries/orchards, the use of resistant rootstocks for propagation, or planting of resistant hybrids for production (Hardham, 2005).

#### Traditional breeding programs for *Phytophthora cinnamomi* resistance

In Europe several first-generation Euro-Asian hybrids have been produced by conventional breeding. Large backcross breeding programs have not been carried out to obtain a nearly wild-type disease-resistant European chestnut, and this may have cost a loss in specific characteristics of the European chestnut. However, many of the obtained hybrids have been successfully used as resistant rootstocks for European varieties, or as nut producing trees for having both resistance to the pathogen and sweet-tasting nuts. The most known example of this is the Euro-Japanese hybrid, CA04 or ‘Bouche de Bétizac’ (BB) developed by INRA (France) in 1962 (Table 2; Breisch et al., 1995). This hybrid became popular for having very large, sweet-tasting nuts and fast production. Cultivar selection has also been extensive in Italy, Spain, and Portugal, and regional favorites are developed mainly from local wild populations with large-caliber nuts.

**TABLE 2:** Summary of efforts to control *Phytophthora cinnamomi*.

Source of resistance/Plant material	Approach for improvement	Resources related to resistance			Current status/Outcomes	References
		Marker type and loci	Gene	Gene function (putative)		
*Castanea crenata*	Controlled crosses with *C. sativa*	NA	NA	NA	F1 hybrids used as resistant rootstocks (e.g., Bouche de Bétizac)	Breisch et al., 1995
*C. crenata*	Controlled crosses with *C. sativa*	NA	NA	NA	53 resistant F1 hybrids (e.g., Colutad)	Gomes Laranjo et al., 2007; Martins et al., 2009
*C. crenata* *C. mollissima*	Controlled crosses with *C. sativa*	NA	NA	NA	SC55, SC914, SC1202 SM904: F1 hybrids with different levels of resistance	Costa et al., 2011; Santos et al., 2015a; Fernandes et al., 2020a,2021a
BC_1_F_1_ *C. dentata* × *C. dentata-mollissima* ‘Nanking’	Study genetic architecture of *PC* resistance; future MAS	SSRs LG_E	NA	NA	NA	Kubisiak, 2010
BC_1_, BC_4_ ‘Nanking’ and ‘Mahogany’		SNPs LG_E	NA	NA	NA	Zhebentyayeva et al., 2014
*C. sativa* × *C. crenata* F_1_ hybrids		SNPs; EST-SSRs LG_E: (1) CC_3129_774 (2) CmSNP00773E (3) CC_48142_849 (4) CmSNP00522E (5) AC_32934_470 (6) AC_36335_960 LG_K: (7) CC_46475_1222 (8) CC_6279_2669 (9) AC_14650_453	NA	(1) Hormone signaling (2) PAF1 protein (3) Resistance protein NDR1/HIN1-Like protein 3 (4) Transport of phospholipids (5) Zinc finger, PHD- type (6) Contains a Myb/SANT-like domain (7) Uncharacterized (8) Cellulose synthase (9) Ribosomal_L6_N	NA	Santos et al., 2017b
BC_1_F_1_ BC_3_F_1_ *C. dentata* × *C. dentata-mollissima* ‘Nanking’ and ‘Mahogany’		SNPs LG_A: hb52208; hb39959 LG_C: nk12394 LG_E: h25723; h54539; hb54410; jb79599; jb32342; jb43327; jb18453; jb13258; nk29352; nk35044; nk19473 LG_K: h31744; hb7814; hb27106	NA	NA	NA	Zhebentyayeva et al., 2019
*C. crenata* *C. sativa* *F1 hybrids*	Identification of resistance genes; future MAS	NA	(1) *Cast_C2CD* (2) *Cast_LRR-RLK* (3) *Cast_ABR1* (4) *Cast_Myb4* (5) *Cast_WRKY 31* (6) *Cast_RNF5* (7) *Cast_PE-2* (8) *Cast_Gnk2-like*	(1, 2) Pathogen recognition (3, 4, 5) Transcription factor (6) Ubiquitination regulator (7) Cell wall modification enzyme (8) Antifungal protein	NA	Santos et al., 2017a
*C. crenata*	Identification of resistance genes; future MAS	NA	*CcAOS*	Enzyme in JA pathway	Overexpression Increased tolerance in *Arabidopsis*	Serrazina et al., 2021

Source of resistance/plant material, approach for improvement, resources gathered, and current status and/or outcomes of these efforts are presented. NA, none applied. LG, linkage group; MAS, marker-assisted selection; JA, jasmonic acid.

In 1925 the first crosses started in France (Camus, 1929) and in 1926 started in Spain by Gallastegui, initiating the hybridization program between *C. crenata* and *C. sativa* (Pereira-lorenzo et al., 2010). After the 1940s, several breeding programs settled across Europe (Schad et al., 1952; Urquijo-Landaluze, 1957; Viéitez, 1960; Molina and Viéitez, 1967; Taveira-Fernándes, 1972; Salesses et al., 1993). Some of these programs obtained hybrid genotypes with low tolerance to cold (Breisch et al., 1995) and poor adaptability to the European environmental conditions in general, mainly because these were F1 hybrids (*C. crenata* × *C. sativa*) with 50% of their genetic information from Japanese chestnuts, a species with low tolerance to cold and drought (Fei et al., 2012). Several clones (111-1, 7521, 2671, and 1483) from Spanish breeding programs developed in the mid-20th century are still widely used as rootstocks for their high tolerance to ink disease and very high rootstock compatibility with fruit varieties (Serdar et al., 2019).

In Portugal, the first interspecific crosses were in 1947 by Bernardino Gomes, who used *C. crenata* (Tamba variety) as pollen donor (Vieira Natividade, 1947; Gomes Guerreiro, 1948, 1957). Later, in the 1990s, Professor Lopes Gomes started a breeding program at the University Trás-os-Montes e Alto Douro developing 53 genotypes resistant to ink disease (Table 2; Gomes Laranjo et al., 2007; Martins et al., 2009). More recently, in 2006, a breeding program was initiated (Costa et al., 2011) from which four F1 hybrids were selected for large-scale propagation due to their ability to multiply and root *in vitro*, field development and *PC* resistance levels (Table 2; Santos et al., 2015a; Fernandes et al., 2020a,2021a).

As a consequence of breeding programs, the introgression of Asian alleles has been reported in a natural *C. sativa* forest (Alcaide et al., 2022). Adult and juvenile (offspring) trees were genotyped and *PC* resistance was detected. Back in the 1940s, *C. crenata* and *C. mollissima* were planted in the studied forest, which justifies the presence of only 70.6 and 28.6% of adults and juveniles, respectively, classified as pure *C. sativa* in this area. Alcaide et al. (2022) also reported more than 40% of juveniles as *C. sativa* × *C. crenata* hybrids and about 10% *C. sativa* × *C. mollissima* hybrids. Ten private alleles to Asian species were found in offspring, eight were exclusive to *C. crenata*, and two were found in *C. crenata* and *C. mollissima* species (Alcaide et al., 2022). The studied forest may benefit from the transfer of alleles involved in ink-disease resistance, and this advantage may be present in other European forests and orchards. However, more forest assessments should be performed to ensure that the European genotypes are not lost over generations.

#### Screening for *Phytophthora cinnamomi* resistant genotypes

With the increasing demand to support and accelerate breeding, phenotyping chestnut genotypes has been performed using different techniques. Symptom severity scales and measurements for subsequent accurate molecular marker: trait associations were reported turning screening for *Phytophthora* spp. resistance more efficient. Either by root inoculation of intact seedlings (Vettraino et al., 2001; Santini et al., 2003; Robin et al., 2006; Jeffers et al., 2009), root inoculation of rooted cuttings (Miranda-Fontaíña et al., 2007) or rooted shoots from *in vitro* culture (Cuenca et al., 2009; Santos et al., 2015a). Also, excised, or intact shoot from seedlings or clones were directly inoculated on its top (Ramos Guedes-Lafargue and Salesses, 1999; Fernández-López et al., 2001; Vettraino et al., 2001; Robin et al., 2006; Miranda-Fontaíña et al., 2007; Cuenca et al., 2009; Santos et al., 2015a). Phenotyping assays in chestnuts are usually performed at leaf-falling time (autumn) and after budburst (spring) (Santos et al., 2015a). According to Santos et al. (2015a), the root inoculation test was the best-resulted method to mimic the infection process in nature. However, this evaluation is expensive, laborious, and sometimes it is not feasible at the population level.

Studies performing the evaluation of responses after root inoculations reported a decrease in the severity of symptoms from the root to shoots. Also, different root-lesion phenotypes were observed depending on genotype susceptibility to the pathogen. The most resistant genotypes can confine or stop the spreading of the pathogen in the roots and from roots to root-collar, unlike the more susceptible ones that usually present their root system majorly affected, and consequently wilting of leaves (Miranda-Fontaíña et al., 2007; Santos et al., 2015a). Furthermore, control plants grow more than those subjected to inoculation (Robin et al., 2006; Miranda-Fontaíña et al., 2007; Santos et al., 2015a), which is expected as one of the consequences of the disease is the reduction of water and nutrient uptake, that consequently affects photosynthetic yield and plant growth (Santos et al., 2015a).

Depending on the phenotyping method (root or excised shoot), different metrics can be used to score the disease damage, and there are dissimilar opinions about which variable should be considered the main discriminator of resistance to *PC*. Previous studies specified the root or collar rot level should be considered the primary discriminator of resistance to *Phytophthora* sp. in chestnut (Robin et al., 2006; Miranda-Fontaíña et al., 2007; Cuenca et al., 2009). Other studies consider plant survival the primary indicator of resistance (Vettraino et al., 2001; Santos et al., 2015a). Santos et al. (2015a) stated the variable ‘days of survival’ was an excellent parameter to define resistance because the difference in the response of the genotypes is accentuated, and the heritability values are high (0.9 ± 0.04). In this work, shoot internal lesion-symptom was evaluated for the first time and it was considered crucial because it showed the advance of the disease lesion from roots and collar to aerial plant organs through the vascular system (Santos et al., 2015a).

#### Improving characterization of *Phytophthora cinnamomi* resistance

After realizing the importance of breeding for ink disease, North American TACF researchers started analyzing their trees from the backcross breeding program for *PC* resistance. In 2010, a preliminary study reported in a single major effect QTL in LG_E that explained more than 30% of the variation in a backcross population (Table 2; Kubisiak, 2010). These findings were later supported by Zhebentyayeva et al. (2014) who identified a major effect QTL for ink disease resistance in the same LG by studying several populations segregating for ink disease resistance (Table 2). In 2015, in Europe, Santos et al. (2015b) developed Simple-Sequence Repeats from Expressed Sequenced Tags (EST-SSR). These, together with Single Nucleotide Polymorphism (SNP) markers, were later used to construct an interspecific linkage map where two QTLs for *PC* resistance were identified (Santos et al., 2017b). The markers associated with QTL in LG_E and LG_K may enclose candidate genes to *PC* resistance, and genes putatively involved with the regulation of gene expression, respectively (Table 2; Santos et al., 2017b).

The arrival of next-generation sequencing revolutionized several research areas, including the detection and validation of genetic markers in wild and hybrid populations. Using GBS, Zhebentyayeva et al. (2019) increased the number of available markers for linkage analysis, mapping 7,715 sequence-based SNPs on eight parental genetic maps. Seventeen QTLs were associated with ink disease resistance on LG_A, LG_C, LG_E, and LG_K (Table 2). The most consistent QTLs were detected on LG_E and LG_K, which overlapped with QTLs previously reported by Santos et al. (2017b). The authors suggest the genetic architecture of *PC*’s resistance in Chinese chestnut × American chestnut hybrid progeny is due to some dominant QTLs together with quantitatively inherited partial resistance conferred by multiple small-effect QTLs (Zhebentyayeva et al., 2019).

In Europe, root transcriptomes of *C. crenata* and *C. sativa* inoculated and non-inoculated with *PC* were compared, resulting in the discovery of 283 differentially expressed genes as candidates for *PC* resistance (Serrazina et al., 2015). In 2017, eight of those genes were selected for further study and by using digital PCR their expression was evaluated in chestnut roots before and during infection (Table 2; Santos et al., 2017a). The authors suggest that European and Japanese chestnuts have the same defense mechanisms to *PC* but with different timings. The upregulation of a set of candidate genes after infection, such as *Cast_Gnk2-like* (anti-fungal function) and *Cast_C2CD* (pathogen recognition protein), suggests that *C. crenata* triggers HR-like cell death. The high expression of these genes in non-inoculated *C. crenata* compared to non-inoculated *C. sativa*, suggests improved constitutive defense mechanisms by the Japanese chestnut (Santos et al., 2017a). Indeed, these hypotheses were confirmed by Fernandes et al. (2021b) at the cellular level, who reported shared host responses in these two species following pathogen challenge (e.g., callose deposition and phenolic-like compounds accumulation). *Cast_Gnk2-like*, was identified as the best discriminator between susceptible and resistant genotypes to ink disease (Santos et al., 2017a), and efforts for the validation of its function in chestnuts are ongoing (Table 2; McGuigan et al., 2020). The gene *Allene oxide synthase* (*CcAOS*), an ortholog of a key enzyme of the JA pathway, was also selected from 2015 transcriptomes (Serrazina et al., 2015). The importance of this gene in plant defense responses against *PC* was demonstrated after being overexpressed in a susceptible *Arabidopsis* ecotype (Ler-0), resulting in a delay of infection progression and an increase in tolerance (Table 2; Serrazina et al., 2021).

Reports on ink disease molecular analysis discussed so far have been focused on root inoculations because it mimics what happens in nature. Saiz-Fernández et al. (2020) presented an alternative by inoculating European chestnut stems and collecting tissues bordering the infection site and away from it. Proteomic, metabolomic, and targeted hormone analysis showed that *PC* led to an accumulation of SA and JA and a massive reprogramming of the chestnut’s proteome. Twenty-five proteins were identified as oppositely regulated in the areas next to and away from the infection site (Saiz-Fernández et al., 2020).

When studying plant-pathogen interactions, the first and most common approach is to unveil the host resistance mechanisms. To date, plant susceptibility (S) genes were only studied in a few woody species, as discussed by Pavese et al. (2021b). S-genes allow the compatibility of the pathogen to the host, facilitating infection. A mutated or lost S-gene may limit the pathogen’s ability to induce host disease. The authors identified and characterized S-genes in *C. sativa* based on sequence homology, functional domain detection, and phylogenetic relationships. Transcript levels of S-genes after pathogen infection (both *PC* and *CP*) were generally higher in *C. sativa* when compared to *C. crenata.* Two genes were selected for future studies on their putative role as S-genes in chestnut-pathogen interactions: *Powdery mildew resistance 4* (*pmr4*) and *Downy Mildew Resistance 6* (*dmr6*) which are suggested to act as negative regulators of SA pathway, consequently leading to susceptibility (Pavese et al., 2021b).

### Genetic transformation as a tool for pathogen control

The emerging progress of genetic transformation systems for chestnuts has been an extremely encouraging story and nowadays genes can be tested for their ability to confer pathogen resistance. The progress of embryogenic regeneration systems for chestnut species [reviewed in Corredoira et al. (2017)] has provided fitting target material for transformation experiments. Carraway et al. (1994) did the first attempt on *Castanea* spp. genetic transformation, however, only obtained transgenic calli by using microprojectile bombardment in cultures of American chestnut. Since then, chestnut researchers have been dedicated to improving genetic transformation of this recalcitrant species. The first report of successful *Agrobacterium-*mediated transformation in *Castanea* spp. showed transgenic European chestnut shoots regenerated from hypocotyls but, transformation efficiencies were very low, and the number of chimeras was high (Seabra and Pais, 1998). This was followed by the transformation of European chestnut somatic embryos that were regenerated into whole plants and micropropagated (Corredoira et al., 2004). The authors recorded a maximum of 25% transformation efficiency after somatic embryo co-culture with *Agrobacterium* liquid suspension for 4 days (Corredoira et al., 2004). The first successful genetic transformed American chestnut was reported in 2006 by co-culturing somatic embryos with *Agrobacterium* liquid suspension (for 1 h) followed by a 2-day desiccation method (Polin et al., 2006). Rothrock et al. (2007) also transformed American chestnut by flooding the embryos with *Agrobacterium* liquid culture while still in semi-solid multiplication media. After these protocols, several works were published on the transformation of chestnut with pathogen resistance genes or to validate gene function. Genetic transformation studies for pathogen control are summarized in Table 3.

**TABLE 3:** Genetic transformation studies performed in European and American chestnuts with the goal of developing pathogen control strategies.

Explant	Approach	Gene (origin)	Gene function	Targeted pathogen	References
*C. dentata* somatic embryos	*Agrobacterium*-mediated transformation	*OxO* (wheat)	Detoxifying enzyme; degrades oxalic acid	*Cryphonectria parasitica*	Polin et al., 2006; Rothrock et al., 2007
					McGuigan et al., 2020
		*OxO* (wheat) (wound inducible promotor)			Carlson et al., 2021
*C. sativa* somatic embryos	*Agrobacterium*-mediated transformation	*CsCh3* (*C. sativa* cotyledons)	Chitinase-like protein; hydrolyses chitin from pathogen’s cell wall	*Cryphonectria parasitica*	Corredoira et al., 2016
*C. sativa* somatic embryos	*Agrobacterium*-mediated transformation	*CsTL1* (*C. sativa* cotyledons)	Thaumatin-like protein; promotes osmotic rupture in the pathogen	*Phytophthora cinnamomi*	Corredoira et al., 2012
*C. dentata* somatic embryos	*Agrobacterium*-mediated transformation	*Cast_Gnk2-like* (*C. crenata* roots)	Antifungal	*Phytophthora cinnamomi*	McGuigan et al., 2020

Initial explant, inserted gene and origin, gene function, and target pathogen are presented.

The rise of genetic transformation had a big impact, but public perception of the use of transgenes is not unanimous. Nowadays researchers are looking more into the use of cisgenes (transgenes from related species) (Corredoira et al., 2012, 2016; Steiner et al., 2017), trying to answer public concerns. Corredoira et al. (2012, 2016) obtained cisgene overexpressing lines with a (1) thaumatin-like protein (*CsTL1*) gene (Table 3; Corredoira et al., 2012); and (2) an endochitinase gene (*CsCh3*) (Table 3; Corredoira et al., 2016). More recently, McGuigan et al. (2020) transformed American chestnut with *Cast_Gnk2-like* (Table 3), a candidate resistance gene for *PC* (Santos et al., 2017a). In McGuigan et al. (2020) the authors also report two alternative methods for transformant selection by using liquid selection medium instead of semi-solid medium like the previously mentioned protocols. After the Agro-kill step, embryos were transferred to temporary immersion bioreactor systems (RITA^®^ bioreactors, Sigma-Aldrich, St. Louis, MO, United States) or We Vitro containers cultivated by Magenta^®^ (We Vitro Inc., Guelph, ON, Canada) where they were intermittently flooded and rocked, respectively. Although the treatments were not significantly different, the liquid medium protocols had more selection efficiency (McGuigan et al., 2020). As the genetic transformation techniques improve, targeted promoters that can replace the most common constitutive promotors also arise. An example of this is the *win3.12* inducible promotor from poplar (*Populus deltoides*), which has positively driven the gene oxalate oxidase (*OxO*) in American chestnut, showing a low level of baseline expression and being only induced by wounding and pathogen infection (Table 3; Carlson et al., 2021).

#### The genetically engineered American chestnut – The Darling 58

North American researchers have allied their breeding efforts to genetic transformation as this can be a quicker way to restoration when compared to just the traditional backcross breeding. In 1990, the TACF New York Chapter and the State University of New York-College of Environmental Science and Forestry (SUNY-ESF) started working on this alternative approach for restoration. A blight-resistant American chestnut tree (Darling 58, named after Herb Darling) was developed by genetic transformation (Polin et al., 2006; Rothrock et al., 2007; McGuigan et al., 2020) by adding to the genome a gene from wheat that encodes for a detoxifying enzyme, oxalate oxidase (*OxO*), to counter the major virulence factor of the pathogen (Powell et al., 2019). Oxalate oxidase degrades oxalic acid, a toxin produced by *CP* during infection (Rigling and Prospero, 2018), limiting the pathogen’s damage without killing it. Currently, these trees are in regulated field plots awaiting deregulation for restoration purposes (Table 1; Newhouse et al., 2020). Crosses of transgenic chestnuts with wild-type American chestnuts are being performed in these plots (Westbrook et al., 2020a). The progeny is 100% American chestnut and approximately 50% of the offspring inherits *OxO* (which is rapidly detected by PCR or enzymatic assays; Zhang et al., 2013a). To increase genetic diversity, up to 4 transgenic chestnuts will be crossed with 1500 wild-type trees over up to 5 generations (Westbrook et al., 2020a). Transgenic pollen can be produced in less than a year (Baier et al., 2012; Pilkey, 2021), which helps expedite the process. Deregulation of the Darling American chestnut represents an important step toward restoring the species. However, public perception of introgressing a transgenic tree into the forest is not unanimous [discussed in Newhouse and Powell (2021)].

### Gene editing

Another exciting news for chestnut genome editing is the first proof of concept of CRISPR/Cas9, where the authors obtained albino plants by inducing a point mutation in *phytoene desaturase* (*pds*), a gene that disrupts chlorophyll biosynthesis (Pavese et al., 2021a). This new approach opens a new path for the functional characterization of genes involved in plant-pathogen interaction. The same research team characterized and selected two S-genes in *C. sativa* after *PC* and *CP* infection (*pmr4* and *dmr6* referred to in Section “Improving characterization of *Phytophthora cinnamomi* resistance”; Pavese et al., 2021a) which are potential candidates for functional characterization *via* CRISPR/Cas9 knockdown. This approach and the study of S-genes might help us understand if *PC* is adapted to the susceptible chestnuts and how it is interfering with their immunity and possibly inducing Effector Triggered Susceptibility.

### Pests – *Castanea* spp. and *Dryocosmus kuriphilus*

**FIGURE 4:**
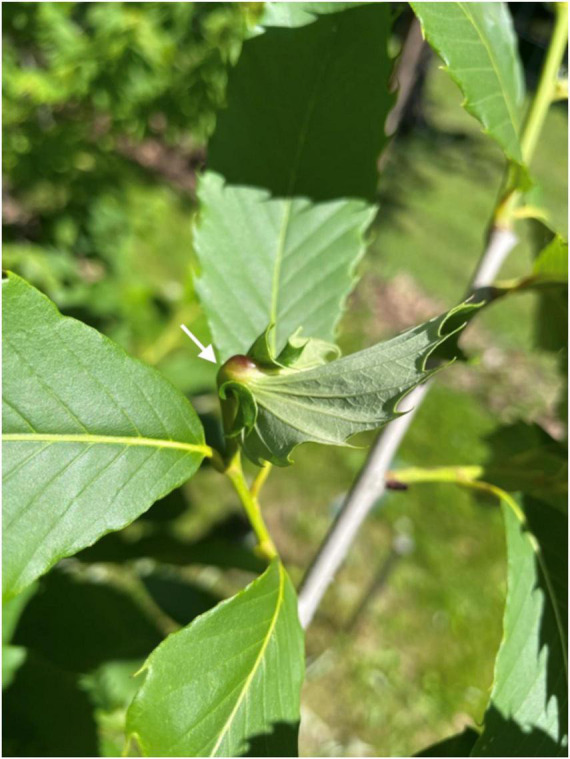
*Castanea* spp. presenting a gall on the leaf (arrow) after *Dryocosmus kuriphilus* infection.

The chestnut gall wasp *Dryocosmus kuriphilus* Yasumatsu, accidentally introduced into Italy and first reported in 2002 (Brussino et al., 2002), represents a limiting pest for the European chestnut, due to the severe yield losses it creates, as *C. sativa* has a low tolerance. Figure 4 shows a chestnut infected by the gall wasp. It quickly spread to all Italian regions and later into the surrounding countries, causing a remarkable decrease in production (−60% in 2014 in Italy). Studies on biological control aimed at introducing the parasitoid wasp *Torymus sinensis* Kamijus, and also the genetic improvement for resistance to the cynipid were promptly started to solve the problem (Marinoni et al., 2020). The susceptibility to the chestnut gall wasp was evaluated in *C. sativa* and hybrid cultivars. Out of 62 cultivars, two *C. sativa*, one *C. crenata*, and four hybrids (*C. sativa* × *C. crenata*) showed total resistance (Sartor et al., 2015).

Resistance to the gall wasp was found in the hybrid cultivar Bouche de Bétizac (BB; *C. sativa* × *C. crenata*) (Dini et al., 2012) and studied by developing genetic linkage maps using a population derived from a cross between BB and the susceptible cultivar ‘Madonna’ (M; *C. sativa*) (Marinoni et al., 2020). The high-density genetic maps were constructed using double-digest restriction site-associated DNA-seq and simple sequence repeat markers. The map of BB consisted of 1,459 loci and spanned 809.6 cM; the map of ‘Madonna’ consisted of 1,089 loci and spanned 753.3 cM. In both maps, 12 linkage groups were identified. A single major QTL (Rdk1) was identified on the BB map, explaining up to 67–69% of the phenotypic variance of the resistance trait. The Rdk1 QTL region includes eleven scaffolds and two candidate genes putatively involved in the resistance (Marinoni et al., 2020). Acquadro et al. (2020) presented *de novo* assembly of the chestnut transcriptome of the resistant Euro-Japanese hybrid BB and the susceptible cultivar M, collecting RNA from buds, at different stages of budburst to investigate the plant response and understand which factors can lead the plant to develop or not the gall, to reconstruct the transcriptome of *C. sativa* buds under biotic stress (i.e., in the presence/absence of the chestnut gall wasp). The two transcriptomes were assembled into 34,081 (BB) and 30,605 (M) unigenes, respectively. The transcriptomes of both cultivars were properly assembled, and while the BB unigenes set was selected for the functional characterization, the M was just used for RNA-seq data analysis, highlighting the presence of 1,444 putative resistance gene analogs (RGAs) and about 1,135 unigenes, as putative miRNA targets. A global quantitative transcriptome profiling revealed some Gene Ontology enrichments as “response to stimulus” and “developmental processes” (e.g., post-embryonic development). Many up-regulated genes appeared to be transcription factors (e.g., *RAV1*, *AP2/ERF*, and *WRKY33*) or protein regulators (e.g., *RAPTOR1B*) and storage proteins (e.g., LEA D29) involved in “post-embryonic development.” Dini et al. (2012) highlighted the occurrence of HR in BB as a response to the cynipid infestation, resulting in cell and larvae death. This fact was confirmed by Acquadro et al. (2020), since more than 100 genes appeared to be associated with “death” and “apoptosis” processes, including genes for HR response. The analysis was able to provide 7k simple sequence repeat SSR and 335k SNP/INDEL markers and generated the first reference unigene catalog for the European chestnut.

Gall wasp, apart from representing a severe constraint factor for the production of chestnut orchards, can also impact negatively the favorable effect of hypovirulence in *Cryphonectria parasitica*-*C. sativa* pathosystem, by the progressive weakening of the trees, caused by intensive attacks of *Dryocosmus kuriphilus*. Blight damage recurrences were observed in different Italian chestnut areas (in Piemonte, Trentino, and Toscana regions which were highly infested by the Chinese wasp; Turchetti et al., 2010). The use of effective biological control of the parasite with parasitoids like *T. sinensis*, is essential for the management of chestnut orchards to allow the survival of the trees and their productivity.

## Abiotic stresses of *C. sativa*

The preponderance of reports on abiotic stresses focuses on the European chestnut. Since the beginning of the millennium and the awareness of climate change scenarios, studies on its impact on the species flourished (e.g., Casasoli et al., 2004; Lauteri et al., 2004). *C. sativa* is susceptible to climate change (Camisón et al., 2020; Castellana et al., 2021), raising the probability of abiotic stress events. Prolonged water deprivation or waterlogging and chill hour reduction, combined with rainfall deficit and extreme summer heat in Europe, may cause and prejudice nut and timber productivity and quality (Vázquez et al., 2016; Castellana et al., 2021). These extreme climate scenarios are predicted to be most frequent in Southern Europe (European Commission, 2022), where chestnut orchards have a great representation (Pérez-Girón et al., 2020). The review in this section will mainly focus on this European area.

Climate change is also predicted to significantly impact chestnut pests and pathogens (Burgess et al., 2017; Bonsignore et al., 2020), leading to differences in disease expressions since new strains and infection mechanisms are more likely to arise.

Three flexible domestication levels of *C. sativa* are considered: fruit orchards, coppice, and natural forests (Eriksson et al., 2005; Gomes-Laranjo et al., 2012). The flexibility among levels is related to the exchange of genetic material between the three types of populations, even though genetic variability is higher in natural forests (excluding the American species) and lower in orchards, which originated from domestication. Genetic variability encloses the capacity to adapt to adverse environmental conditions, maintaining homeostasis and reproduction, and the potential to evolve. Changes in morphology and physiology in response to abiotic stresses involve complex molecular processes under genetic and epigenetic control. Besides knowledge at the physiological level, knowledge of the response to the stress at the genetic and molecular level in detail is essential to drive efficient tolerance to threatened species.

### *C. sativa* natural adaptation to different environmental areas

The genetic resources of *C. sativa* natural populations represent the existing variation in adaptive traits (Eriksson et al., 2005). They may be the starting material for breeding programs to address tolerance to abiotic stress in elite varieties. Many studies characterize *C. sativa* ecotypes from the Iberian Peninsula, Italy, Greece, and Turkey (e.g., Villani et al., 1991; Eriksson et al., 2005; Fernandez-Lopez et al., 2005; Pereira-Lorenzo et al., 2011; Míguez-Soto et al., 2019; Castellana et al., 2021).

The environment influences adaptative traits such as annual biomass production, juvenile phenology growth, water use efficiency (WUE), and carbon isotype discrimination (Δ). European chestnut is a temperate-climate tree that requires relatively cool winters for dormancy and then warmer temperatures in spring, allowing physiological and phenological development as bud break, flowering, fruit set, and maturation (Gomes-Laranjo et al., 2012). Phenology depends on temperature, nutritional state, photoperiod, hormones, phytochromes, and others and can represent seasonal and environmental adaptation (Santamaría et al., 2009). WUE is the ratio of plant carbon (C) gain to water loss and is inversely related with Δ, which is affected by CO_2_ assimilation and stomatal conductance (g_s_) (Lauteri et al., 2004). From research mainly on non-cultivated populations, several European chestnut ecotypes are adapted to different climates, corresponding to different evolutionary pressures in the genome. In the Iberian Peninsula, Italy, Greece and Turkey, and based on morphological, physiological and/or molecular markers’ studies (SSRs, EST-SSRs), hotter Mediterranean regions with lower water availability or drought drove populations to xeric ecotypes (early phenology, slow growth, high root development, high Δ, low WUE and longer juvenile periods). In comparison, populations in colder Atlantic regions with more water availability are mesophytic or mesic (later phenology, higher growth, low Δ) (Lauteri et al., 2004; Fernandez-Lopez et al., 2005; Gomes-Laranjo et al., 2012; Míguez-Soto et al., 2019; Camisón et al., 2021). Lauteri et al. (2004) suggest that mesic ecotypes respond strongly to favorable climatic conditions by increasing growth, while xeric ecotypes respond slowly to reduce the risk of damage after drought (e.g., xylem embolism, C starvation). This is in accordance with Pérez-Girón et al. (2020). They compared essential physiological parameters in orchards from two regions of the Iberian Peninsula: northern Spain and Portugal, and southern and central Spain. The authors found the highest annual photosynthesis rate and net primary production (net C stored after respiration and transformed into biomass) in the northern ecosystems. Water availability and temperature were the climatic variables that most influenced the two parameters.

Different European chestnut ecotypes may have origin in an overall high genetic diversity (Villani et al., 1991; Pereira-lorenzo et al., 2010; Cuestas et al., 2017; Poljak et al., 2017; Alessandri et al., 2020; Bouffartigue et al., 2020). Dinis et al. (2011a) and Pereira-Lorenzo et al. (2011) found, with the use of SSR markers, that the diversity in chestnut orchards was greatly due to hybridization and discretely due to mutations. *C. sativa* genetic diversity, with many alleles and a high level of polymorphism and heterozygosity (Casasoli et al., 2006), provides gene pools useful for establishing future conservation strategies. The ecotypes’ traits correspond to adaptations that have the potential to be introgressed in threatened chestnut stands, potentially providing adaptation to climate change.

### *C. sativa* response to drought

Most of the reports about *C. sativa* diseases related to abiotic stress describe the response to drought. *C. sativa* is strongly represented in the European Mediterranean area, which has been experiencing long and dry summers (high temperatures and low precipitation levels) with increasing drought conditions, causing a negative impact on *C. sativa* survival, productivity, and biodiversity (Ciordia et al., 2012; Alcaide et al., 2019; Castellana et al., 2021). *C. sativa* tree growth may be severely affected when the drought period is higher than two consecutive months (Menéndez-Miguélez et al., 2015), with most probable negative consequences on the development of leaves and fruits (Dinis et al., 2011a). Drought response is complex to analyze, as it may be influenced by population history, frequency of drought events, and phenotypic plasticity (Casasoli et al., 2006; Alcaide et al., 2019; Müller and Gailing, 2019).

#### Physiological and biochemical responses to drought

Martínez-Sancho et al. (2017) consider *C. sativa* a relatively anisohydric species in the physiological responses to high temperature and drought, meaning that stomata closing is not readily achieved after water deprivation unless under severe drought conditions. Low water potentials (Ψ) in seedlings resulted in an overall native loss of hydraulic conductivity and probable vessel embolism, accompanied by height and stem diameter decrease compared to controls. The authors suggest that the hydraulic conductivity can be potentially recovered in the next growing season with new earlywood vessels and xylem renovation.

Maurel et al. (2004) report that g_s_, transpiration rate (E), hydraulic conductance (K) from soil to leaf, leaf Ψ, and root biomass decreased in *C. sativa* subjected to drought, whereas abscisic acid (ABA) concentration in xylem increased. The authors also showed that g_s_ was regulated by the root-sourced ABA and by hydraulic signals, namely the relative sap flux from root to leaves. Leaf transpiration is an essential factor in establishing Ψ, or the flow of water from the soil to the roots, stems, leaves (stomata) and atmosphere, with the purpose of mineral uptake and regulation of leaf temperature. During water deprivation, E became seriously compromised, contributing to a significant decrease in overall plant metabolism and productivity (Gomes-Laranjo et al., 2012).

Ciordia et al. (2012) and Gomes-Laranjo et al. (2012) studied progenies of *C. sativa* cultivars (seedlings) from two areas in the Iberian Peninsula, North (Asturias and Galicia, with mesic or moderately humid environment) and Central/South (Canary Islands and Andalusia, with xeric and drier environment). Merging the results from both studies, restricted water supply reduced the Ψ, K (especially in xeric plantlets), CO_2_ assimilation rate (*A*), *E*, *g*_s_ and, consequently, photosynthetic efficiency. Gomes-Laranjo et al. (2012) associated the reduced efficiency of Photosystem II (PII, low *F*_v_/*F*_m_) with an internal CO_2_ concentration increase (Ci) and lower C assimilation, especially in Northern plant leaves. During water deprivation there is a need to reduce light absorption to avoid heat accumulation, resulting in the reduction of PSII efficiency or even photooxidative reduction in extreme conditions. An expected consequence is a reduction of growth (height and dry weight, except stem diameter) (Ciordia et al., 2012). Also, both studies found a reduction of leaf area, number (with no leaf fall) and sprouting, attributed to lower absorption of nutrients. The morphology of the leaves suffered modifications, with an increase in leaf lobation, resulting in a smaller boundary layer and more efficient heat exchange. The root:shoot ratio increased due to biomass distribution changes in response to the low water content in the soil, promoting root biomass that may improve the capacity to absorb water (Ciordia et al., 2012). The same authors consider that the north cultivars are more tolerant to drought than the Central ones, as the first demonstrated a better ability to recover Ψ after re-watering. Both mesic and xeric groups demonstrate phenotypic plasticity that is consistent with the genetic variation found using SSR and EST-SSR (Martin et al., 2010; Pereira-lorenzo et al., 2010), providing such stands the potential to respond to drought stress (Ciordia et al., 2012).

**FIGURE 5:**
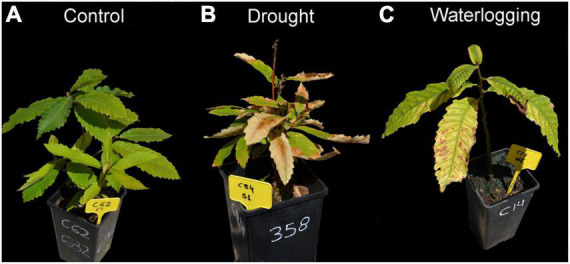
*Castanea sativa* seedlings under regular watering **(A)**, drought **(B)**, and waterlogging **(C)**, from Camisón et al. (2020). Control plants present green and turgid leaves. Leaves of drought plants present wilting and some may fall. Dieback can be observed. Leaves of plants with waterlogging present chlorosis, chlorophyll degradation in the central part of leaves, necrotic borders and senescence.

Camisón et al. (2020) (Figure 5) found that in drought-tolerant *C. sativa* seedlings (of the xeric ecotype from central Spain) the *g*_s_ was close to zero, associated with a decrease in relative water content (RWC), height and weight loss, increase in stem diameter, leaf wilting with occasional drop and plant dieback. Stomatal closure was associated with the reduction of A and soluble sugar accumulation in leaves, which may impair C supply. The authors suggest that soluble sugar accumulation in leaves and stems may have a role in plant osmoregulation. A decrease in leaf biomass was accompanied by an augment in nitrogen (N) levels in leaves due to N transport from senescent to green leaves. The peak of soluble sugar levels in leaves and stems coincided with the highest reduction in starch levels, probably due to starch mobilization as a source of soluble sugars for cell metabolism, osmotic adjustment, and consequent xylem vessel water refilling after drought-induced embolism. High respiration levels in stressed plants are related to the metabolism of soluble sugars to counteract the stress. The authors did not observe changes in total carbohydrate content or C starvation attributable to drought-tolerant species in non-extreme drought conditions.

Economically important chestnut orchards for nut production in Southern Europe are distributed through regions of provenance, many with several decades old, and established recurring to grafting without a conscient concern about the effects of global climate change. Camisón et al. (2021) analyzed if drought tolerance in *C. sativa* could be improved using drought-tolerant scions and rootstocks from xeric populations (X) from southern Spain, in comparison with drought-sensitive scions and rootstocks from humid populations (H, with mesic ecotype) from northern Spain, based in the research of Alcaide et al. (2019). Grafted and non-grafted 2 years old plants were used in the drought treatments. When X scions or rootstocks were used in these conditions, budbreak occurred earlier, and higher *g*_s_ and lower plant mortality were observed. *C. sativa* families of X origin advance budbreak phenology and may be used to induce early flushing in scions of H origin. Benefits could be attributed to the use of X rootstocks to advance budbreak in mesic areas, especially if enhanced growth, flowering, and fruit production are obtained. Grafted plants with X rootstocks (H/X and X/X) showed higher *A*, *g*_s_, *F*_v_/*F*_m_ of PII and leaf RWC than plants with H rootstocks (X/H and H/H). Rootstocks from xeric areas increase drought tolerance and survival of the more drought-susceptible material of mesic origin. On other hand, H used as rootstock lead to minimum values of leaf RWC and *F*_v_/*F*_m_ levels, confirming its susceptibility to drought. Concerning scion, grafts with X rootstock wilted less than grafts with H rootstocks. Scions of xeric origin may also have the potential to improve drought-susceptible rootstocks. Grafting may be implemented as an adaptative tool to surpass climate change’s effects (Camisón et al., 2021).

Camisón et al. (2021) also analyzed constitutive and drought-induced hormones [ABA, SA, JA, its conjugate (+)-7-iso-jasmonoyl-L-isoleucine (JA-Ile)], and the amino acid proline in leaves and roots of grafted and non-grafted plants of X and H origin. Before drought induction, in watered plants, leaf ABA and proline contents were higher in X than in H plants, in non-grafted and grafted material. The constitutive higher levels of ABA in X material may have contributed to lower *g*_s_ and the delay in plant dehydration in X rootstocks after drought treatment (Allario et al., 2013). The same rationale may be applied to proline, which may have conferred to X rootstocks a more effective osmotic adjustment when drought was applied (Amudha and Balasubramani, 2011). After drought treatments, ABA and proline levels increased in leaves and roots of all materials. H/H plant presented the highest levels of ABA in roots and proline in leaves, which points to extreme stress. SA levels were higher in H, H/H and X/H plants. SA increase along with ABA has been reported in citrus response to drought (Santana-Vieira et al., 2016; Neves et al., 2017). JA-Ile level was higher in leaves, especially in H/H plants. JA-Ile in leaves under drought stress, allied to ABA, is involved in stomatal closure modulation (de Ollas et al., 2013). JA-Ile and JA levels in roots generally decreased, and the lowest value was found in H and H/H plants. More studies are needed to understand the hormone crosstalk in *C. sativa* response to drought.

#### Genomic aspects of drought response

Casasoli et al. (2004) performed a QTL analysis for three adaptative traits (bud burst, growth, and Δ), for 3 years, in an F1 progeny of *C. sativa* originated from two Turkish populations adapted to drought (female parent) and humid (male parent) environment. Thirty-five distinct QTLs were identified for phenology, 28 for growth, and 17 for Δ. The authors report phenotypic correlations and co-localization among QTLs for the three adaptative traits related to the genetic adaptation of the female parent to drought. Moreover, the adaptative traits seem to be regulated by several genes or gene groups of low and moderate effects, suggesting that the adaptation, and consequently the response to abiotic stress at the genetic level is highly complex in chestnuts.

Santamaría et al. (2011), after analyzing the transcriptome of dormant and non-dormant tree buds in *C. sativa* trees of Asturias (North of Spain), suggest that bud dormancy is associated with abiotic stress tolerance. There was a high representation of genes involved in low-temperature stress and dehydration protection of cellular structures: Late embryogenesis abundant proteins (*LEA*), including Dehydrins and Em protein, Heat shock proteins (*HSP*), and transcription factors that control the expression of HSPs [reviewed in Kalemba and Pukacka (2007)]. Also, galactinol synthase (*GOLS*) and Raffinose family oligosaccharides (*RFOs*) are involved in desiccation tolerance through protection to oxidative damage (Vinson et al., 2020).

Alcaide et al. (2019) quantified drought response in populations of *C. sativa* localized in contrasting environment regions of the North (lower average temperature and higher precipitation level) and South (higher average temperature and lower precipitation level) of Spain. In 1-year-old seedlings from selected trees, they found a direct correlation between leaf wilting and resprout with survival, indicative of drought tolerance. Individuals from populations in the South with xeric ecotype, thriving in severe drought conditions, were selected as a drought-tolerant resource. Allied to the data on phenotypic tolerance to drought, EST-SSR MAS permitted separation of North and South populations. Four markers were classified as significantly involved in the differentiation of *C. sativa* individuals to drought tolerance (Table 4). *FIR080* showed one allele for drought susceptibility and may correspond to a *Ricin B-like lectin EULS3*, involved in drought stress response through stomatal closure in *Arabidopsis thaliana* (van Hove et al., 2015). *VIT057* corresponds to *Ethylene-responsive transcription factor ERF017*, may act as a transcriptional activator and may be involved in gene regulation by stress factors^1^. *GOT045*, a *probable E3 ubiquitin-protein ligase*, may be involved in regulating ABA-mediated drought stress through ubiquitination (Lee and Kim, 2011; Seo et al., 2012). *FIR059* is putatively linked to the RH7 gene of the DEAD-box-RNA helicase family, which has been implicated in RNA processing and related to abiotic stress responses (Kim et al., 2008). Three alleles of *FIR059* were linked to drought-susceptible individuals, while two alleles were linked to drought-tolerant ones. *FIR059* is pointed as the best marker to identify putative drought-tolerant unstressed trees.

**TABLE 4:** Summary of the tolerance to abiotic stresses in *Castanea sativa* and *C. dentata*.

Abiotic stress/Source of tolerance	Approach for improvement	Resources related to resistance			References
		Marker type and loci	Gene	Gene function	
**Drought**					
*Castanea sativa* forest populations, xeric ecotype, Hervás (Central Spain)	NA	NA	NA	NA	Camisón et al., 2020
*Castanea sativa* forest populations, xeric ecotype: Constantina, Sierra Norte (South Spain), Hervás (Central Spain), Holomontas Hortiatis (North Greece), Bursa (Northeast Turkey)	Identification of tolerance genes	EST-SSR MAS: *FIR080*, *VIT057*, *GOT045*, *FIR059*	*Ricin B-like lectin EULS3; Ethylene-responsive transcription factor ERF017; probable E3 ubiquitin-protein ligase; RH7* gene of the DEAD-box-RNA helicase family	Stomatal closure; transcriptional activator regulated by stress factors; regulation of ABA-mediated drought stress; RNA processing related to abiotic stress responses.	Alcaide et al., 2019; Castellana et al., 2021
*Castanea sativa* forest populations, xeric ecotype, Constantina (South Spain)	Future grafting: rootstocks and scions	NA	NA	NA	Camisón et al., 2021
*Castanea dentata* forest site, Reedsburg, WI, United States	Future breeding for introgression of trait in hybrids of *C. dentata* × *C. mollissima* resistant to *CP*	NA	NA	NA	Bauerle et al., 2006
NA	Future seedling inoculation with ectomycorrhizal fungi	NA	NA	NA	Aryal, 2017
**Heat and cold**					
n.a.	Identification of tolerance genes	NA	Small heat-shock protein *CsHSP17.5*	Prevent irreversible aggregation reactions between stress-labile proteins, maintaining the cytosolic proteins soluble	Soto et al., 1999
**Heat**					
*Castanea sativa* forest populations, xeric ecotype: Almería (South Spain)	Identification of tolerance genes	EST-SSR: *VIT099*, *POR016*	*NAC domain-containing protein78*; heat shock protein 70k (*HSP70*)	Regulation of flavonoid biosynthesis and 20S and 26S proteasomes in response to photooxidative stress; involved in stomatal closure and modulation of ABA-dependent physiological responses that may result in, e.g., thermotolerance	Dorado et al., 2022
NA	Future soil fertilization with Si	NA	NA	NA	Gomes-Laranjo et al., 2018

Sources of tolerant material, genomic data and possible solutions for stress mitigation are presented. Only references directly related to the respective stress are considered. NA, none applied.

Castellana et al. (2021) associated *C. sativa* EST-SSR markers previously related with drought stress (Martin et al., 2010; Alcaide et al., 2019) with xeric or mesophytic natural populations in Spain, Greece and Turkey. Those EST-SSRs differentiated three genetic clusters: group I form areas with low precipitation and high temperatures along the year (Table 4); group II with low temperatures and low precipitations; and group III with moderate-low temperatures and high precipitations. Relations were found between climatic variables and alleles in the locus *FIR059* above mentioned: allele 152 was associated with heavy rain, allele 181 with warm and dry areas, and allele 185 with mild temperatures. Moreover, alleles 152 and 176 were associated to drought-tolerant plants, while allele 160 was linked to drought susceptibility.

### *C. sativa* response to waterlogging

Global climate change will cause, among others, extreme rainfall events with a higher probability of long-term waterlogging in winter and spring, and short-term flooding events during summer (Christensen and Christensen, 2003; Kundzewicz et al., 2005). As *C. sativa* naturally grows on well-drained mid-sloped soils, it has a low tolerance to waterlogging (Glenz et al., 2006). Chestnut orchards established in floodplains can be severely affected by soil flooding, and there are still few studies that characterize the species response to this abiotic stress. Camisón et al. (2020), already mentioned before for analyzing drought stress in *C. sativa*, also analyzed waterlogging effects. The two stresses caused some analogous effects on 1-year-old seedlings with progeny from central Spain, with xeric ecotype: reduced *g*_s_, *A*, and growth. The main negative effect of waterlogging in trees is oxygen deprivation in roots (hypoxia, Kreuzwieser and Rennenberg, 2014), causing a decrease in root hydraulic conductivity, xylem sap flow, and phloem transport. Consequently, the first responses to waterlogging include stomatal closure, followed by a decrease in net CO_2_ assimilation and transpiration. The decrease in g_s_ was not associated with low water content in soil/roots, and Camisón et al. (2020) point to the involvement of chemical signals that regulate *g*_s_ in waterlogged plants.

**FIGURE 6:**
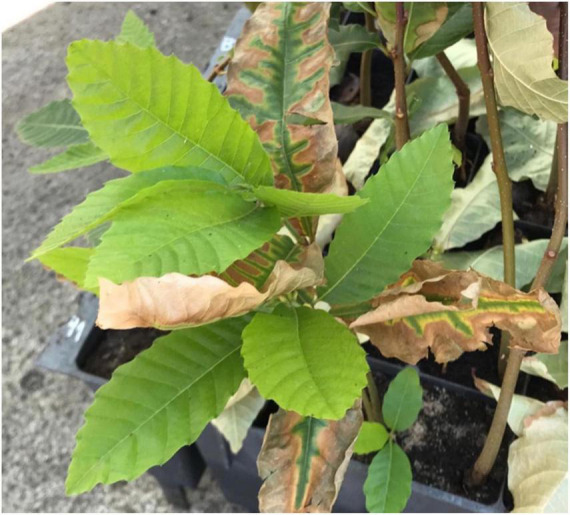
*Castanea sativa* seedling under heat stress conditions described in Dorado et al. (2022).

Contrary to *C. sativa* plants with drought stress, lower N content and C/N ratio in leaves of waterlogged plants were observed (Camisón et al., 2020; Figure 6). This can also be attributed to the disturbances in CO_2_ processing and N uptake by roots. There was also an initial augment of soluble sugar content in all tissues (glucose and sucrose) in waterlogged plants and later an accumulation of starch in stems and roots. This was attributed to an active allocation of C for reserve formation, given the low A rates, and the inability of *C. sativa* to use carbohydrates for respiration and growth during waterlogging. Typically, susceptible plants decrease the activities of key enzymes for glycolysis in leaves and roots during the stress, not stimulating fermentative pathways as an alternative to producing energy (ATP) (Kreuzwieser and Rennenberg, 2014). Although and unexpectedly for susceptible plants, chestnuts formed aerenchyma at the root collar, pointing to the ability of the use of soluble sugars as C sources. When compared to drought-stressed plants, reduced respiration rates in waterlogged chestnuts, were attributed to a low use of soluble carbohydrates, as already mentioned. Another effect in *C. sativa* waterlogged plants was chlorophyll degradation (Camisón et al., 2020). This effect, allied to soluble sugar accumulation in leaves, may have resulted in A reduction. A visible related symptom in waterlogged plants was leaf shedding and chlorosis.

Camisón et al. (2020) consider that the susceptibility of *C. sativa* to waterlogging is related to the residual use of non-structural carbohydrates and the active allocation of C to reserve formation, resulting in an overall dropdown of net primary production and growth. Stress-sensitive trees cannot maintain carbohydrate availability resulting in the decrease of anabolic processes and the dieback of stressed tissues (Kreuzwieser and Rennenberg, 2014).

As drought stress response, the waterlogging stress response, is a highly complex process at the molecular and metabolic levels. The physiological adaptations of trees to waterlogging have a lack of data and data interpretation at the -omics level to advance in the understanding of the tolerance processes that serve as guidance to tree improvement programs.

### *C. sativa* response to heat, cold and salt stress

Soto et al. (1999) describes *C. sativa* response to heat, cold and salt stress. The study focuses on a small heat-shock protein (sHSP) gene purified from mature *C. sativa* cotyledons, named *CsHSP17.5*. sHSP are stress-inducible proteins that can prevent irreversible aggregation reactions between stress-labile proteins, maintaining the cytosolic proteins soluble. They are also denominated ATP-independent molecular chaperones (Santhanagopalan et al., 2015). *CsHSP17.5* was overexpressed in *Escherichia coli* and improved viability under heat stress at 50°C and cold stress at 4°C. On the other hand, *C. sativa* seedlings under 1 year-old were subjected to heat-stress treatments (32°C or 40°C and 80% of relative humidity for 8 h), cold-treatments (4°C for up to 4 weeks) and salt-stress treatments (watering with 200 mM NaCl for up to 48 h). In vegetative organs of chestnut plantlets, transcripts hybridized with a *CsHSP17.5* cDNA probe in heat, cold, but not in salt stress. Despite the complexity and polygenic response of plants to abiotic stresses, *CsHSP17.5* is an interesting candidate to consider for biotechnological approaches to chestnut improvement to heat, drought (to which heat is mostly allied) and cold stresses (Table 4).

In a just released report (Dorado et al., 2022) where heat stress was tested, a significant increase in the osmolyte proline was observed in *C. sativa* leaves from humid forests, less thermophilic-tolerant. No variation was noted in *C. sativa* of xeric origin. Two EST-SSR markers are suggested to be included in the early selection of tolerant chestnuts to heat stress: locus *VIT099* (NAC domain-containing protein 78) and *POR016* (Heat shock protein 70 k) (Table 4 and Figure 6).

### Cumulative abiotic and biotic stresses may impact chestnut populations

Natural populations of European chestnut seem to adapt better to dry climates than to waterlogging (Vázquez et al., 2016; Camisón et al., 2020). Waterlogging is especially challenging for chestnuts susceptible to *Phytophthora* spp. growing in favorable edaphoclimatic conditions, as the pathogen causes high mortality in those scenarios (Vázquez et al., 2016). Drought and waterlogging negatively influence plant growth and induce loss of plant vigor, which causes susceptibility to pests and diseases (Maurel et al., 2001; Gomes-Laranjo et al., 2004, 2012; Dinis et al., 2011b).

*C. sativa* infected with hypovirulent strains of *CP*, located in the Central Eastern Alps in Italy, showed increased mortality caused by a synergistic effect of blight infection and drought stress (Waldboth and Oberhuber, 2009). The trees were standing in regions with low precipitation during winter and high temperatures in spring and summer. Gomes-Laranjo et al. (2004) suggest that *PC* infectious capacity may increase with drought and heat, as plants have superior water uptake by roots and higher E, and the root damage caused by the pathogen action may accelerate plant decay. Moreover, Vázquez et al. (2016) report that if drought-stressed *C. sativa* seedlings are infected with *PC* and drought conditions are prolonged or waterlogging is applied, the mortality caused by the pathogen is highly significant.

Camisón et al. (2019) made an interesting study in which they assessed the drought stress tolerance in offspring of *PC*-infected *C. sativa* trees (from a forest in Southwest Spain of the xeric ecotype). Despite the increased tolerance to the pathogen in 1 year-old plants, suggesting the response was triggered in the subsequent generation, increased tolerance to water stress was not verified, therefore the infection did not influence dehydration stress memory.

Asian Chestnut species have different edaphoclimatic growth conditions when compared to *C. sativa*, being adapted to more humid environments. Pereira-lorenzo et al. (2010), Fernandez-Lopez (2011) and Vázquez et al. (2016) reveal that Euro-Asian hybrids bred for *PC* resistance, are susceptible to the frequently occurring summer water stresses in Southern latitudes of Spain. Deep studies on the impact of abiotic stresses in those hybrid clones cultivated in Europe, compared to *C. sativa*, need to come to light.

Contrary to the chestnuts in Europe, a different positive scenario occurs for the *C. dentata* x *C. mollissima* blight-resistant hybrids of the backcross breeding program previously mentioned. These hybrids, ∼94% *C. dentata* and 6% *C. mollissima*, are indistinguishable from the original *C. dentata*. Bauerle et al. (2006) studied the ecophysiological response of *C. dentata* seedlings to drought. They found that seedlings can decrease *g*_s_ and maintain Ψ, permitting a balance between transpiration and photosynthesis rate (A), and consequently increase WUE and C gain. The results point to drought tolerance of *C. dentata*, and if the trait is introgressed in blight-resistant hybrids, the reintroduction of the species in climate change scenarios predicted for the native *C. dentata* sites of the eastern United States may be facilitated (Brown et al., 2014) (Table 4).

### Mitigation of abiotic stresses

#### *Castanea mycorrhizae* as sustainable helpers in the defense against abiotic stress

Arbuscular mycorrhizal fungi (AMF) are part of the soil microbiome and the symbiotic association with the rooting system of forest species is essential to plant nutrition and growth. AMF also helps recovery of abiotic stresses in plants, such as drought, flooding, extreme temperatures, salinity, heavy metals (Diagne et al., 2020), and biotic stresses caused by soil pathogens such as *PC* (e.g., in *C. sativa*, Branzanti et al., 1999). Equally important is that AMF improves soil aggregation and establishes interactions with beneficial soil microorganisms, upgrading the ecosystem (Diagne et al., 2020).

The AMF increases plant tolerance to extreme temperatures (heat and cold) by maintaining water and nutrient uptake, photosynthesis capacity, alleviating oxidative damage, increasing osmolytes’ level, and consequently, improving growth. In plants subjected to high salinity, AMF enhances water and nutrient uptake, the accumulation of osmoregulators such as proline and sugars, and ionic homeostasis, including reducing Na^+^ and Cl^–^ uptake (Diagne et al., 2020).

*C. dentata* × *C. mollissima* seedlings inoculated with ectomycorrhizal fungi (*Pisolithus tinctorius* and four *Rhizopogon* species), and subjected to drought, recovered faster than non-inoculated plants after rewetting (Aryal, 2017) (Table 4). The author based the conclusions on chlorophyll fluorescence levels (*F*_v_/*F*_m_).

At the genetic level, *C. sativa* in symbiosis with the AMF *Pisolithus tinctorius* may regulate the expression of genes related to abiotic stress (Sebastiana et al., 2009), as *Cystatin*, coding a cysteine protease inhibitor that may limit stress-induced proteolysis (Kunert et al., 2015), and *Lipid transfer/seed storage/trypsin-alpha amylase inhibitor*, corresponding to soluble proteins that facilitate the transfer of fatty acids (García-Olmedo et al., 1995). Curiously, Pernas et al. (2000) reported that non-mycorrhized *C. sativa* plantlets subjected to cold-, saline- and heat-shocks strongly upregulated a cystatin in roots and leaves, pointing to a relevant role of cystatins in abiotic stress mitigation.

#### Silicon fertilization as a prophylactic agent against abiotic stresses

Silicon (Si) is being studied as an ally for the resilience of crops to abiotic and biotic stresses, such as drought, high temperature, cold, salinity, heavy metal toxicity [reviewed in Choudhury et al. (2020)] and fungi, bacteria and herbivores [reviewed in Ma (2004)]. Recently it has been found that the described effects of Si in the alleviation of abiotic and biotic stresses are related to the modulation and regulation of genes involved in photosynthesis, transcription, water transport, phenylpropanoid- and ABA-dependent pathway (Manivannan and Ahn, 2017).

Zhang et al. (2013b) evaluated the foliar application of Sili-K solution on leaves after short but intense water deprivation, in potted plants of the genotype Ca90 (*C. sativa* × *C. crenata*, *PC*-resistant). They observed mitigation in heat stress: the improved leaf growth was related to the increase in A. Si may have increased g_s_ and E, allowing more CO_2_ to enter in the leaf, decreasing leaf temperature and increasing the transport of nutrients to leaves, respectively. The improved A was also associated with higher *F*_v_/*F*_m_ of PSII and chlorophyll a and b content, suggested by a higher proportion of stacking in thylakoid membranes with less photo inhibitory damage. Moreover, Si may participate in the thermal stability of phospholipids in membranes. However, the foliar application of Si also increases the susceptibility to drought. Si application in potted plants with restricted space for root growth resulted in a lower concentration of soluble sugars, which was related to a lower osmotic pressure that may have decreased the cellular turgor and lowered leaf sap osmotic pressure, causing the plant to lose water more quickly (Zhang et al., 2013b).

Gomes-Laranjo et al. (2018) noted that chestnut roots have a good ability to absorb and accumulate Si in the plant. Two-month-old *C. sativa* were subjected to high temperatures and the soil was fertilized with Si. The authors observed the deposition of phytoliths in leaves and conducting vessels, which was associated with the increase of chlorophyll a/b and carotenoid content, and with the protection of the photosynthetic apparatus from oxidative damage. Also, a lower decrease in A and the increase of WUE was reported when compared to non-fertilized plants. The presence of phytoliths was also involved in the regulation of the stomatal movement, increase of cuticle thickness, and decrease in water loss, related to the lower values of *E* and *g*_s_. Moreover, Si fertilization resulted in plants with a higher level of unsaturated lipids, improving membrane stability and integrity under high temperatures (Gomes-Laranjo et al., 2018) (Table 4).

In a later report, (Carneiro-Carvalho et al., 2019) verified the recovery capacity of 2-month-old *C. sativa* plants after a turnover from a high temperature to an adequate temperature. Si-fertilized plants showed a better recovery rate when compared to non-fertilized plants, reducing the oxidative damage and improving osmoregulation through the increase in the activity of antioxidant enzymes and metabolites such as catalase, ascorbate peroxidase, peroxidase, and phenols. These may have contributed to the reduction of electrolyte leakage, lipid peroxidation, and reactive oxygen species content.

Si fertilization seems to be absent of negative secondary effects for the environment or human health, making it sustainable for agriculture purposes.

## Conclusion and future perspectives

Although many biotic and abiotic stresses threaten chestnuts, many efforts are being developed to overcome the challenges and save these important species. The incidence of *PC* and *CP* infections might become more severe in the context of climate change with the possible rise of new strains. Increasing the number of genetic and genomic resources combined with the development of high-throughput phenotyping technologies will help us reach marker-assisted selection (MAS). Screening individual trees within a breeding population and identifying the genetic basis of important traits should become faster, possibly reducing the number of BC needed in traditional breeding programs. The *de novo* transcriptome assembly provided a significant contribution, however, future studies can now rely on genomes of *C. mollissima* (Xing et al., 2019; Staton et al., 2020), *C. crenata* (Shirasawa et al., 2021), and *C. dentata* (Sandercock et al., 2022).

The potential of new approaches such as CRISPR/Cas systems in trees can result in desired changes such as introducing a gene of interest or targeted gene(s) knockouts of undesirable genes in plants (Limera et al., 2017). Added to gene silencing, the activation of genes and overexpression of proteins can be achieved by CRISPR/dCas9 (nuclease-dead Cas9), conferring new possibilities for gene functional analysis and characterization (Moradpour and Abdulah, 2020). The integrated use of these technological developments with biotic and abiotic stresses will help expand our capabilities of response to chestnut challenges.

The next steps for chestnut breeding may include improving resistance to both pathogens and looking for durable resistance. After screening Asian-American hybrid seedlings with improved blight resistance to *PC*, TACF found out that many of their hybrids were resistant to the oomycete (Zhebentyayeva et al., 2014, 2019). Researchers now believe that traditional breeding could be used to combine resistance (Steiner et al., 2017). It is also suggested that this could be achieved by stacking multiple resistance genes now that chestnut genetic transformation techniques have been optimized (Powell et al., 2019; McGuigan et al., 2020). There is also an increasing interest in cisgenes possibly because the deregulation process would be more straightforward. Moreover, the public perception may not be as fragmented as it is on the use of transgenics. Stacking cisgenes related to different defense mechanisms can help enhance resistance by different mechanisms. However, it is not expected that any of these genes will confer full resistance to the pathogens. For example, *OxO* is still the gene demonstrating the highest levels of resistance to blight disease (Steiner et al., 2017). Thus, we should just take advantage of all tools and combine transgenes and cisgenes.

The future roadmap for *Castanea-*pathogen studies may benefit from exploring the pathogen’s virulence/avirulence factors specific to chestnut interactions. The available genomes of *PC* (Engelbrecht et al., 2021) and *CP* (Crouch et al., 2020) may bring some insight. Additionally, dual RNA sequencing enables the determination of responses and changes in the cellular networks of both organisms, which has already helped understand ink disease in other plants (Meyer et al., 2016; Evangelisti et al., 2017). Identifying *CP* molecular weapons might increase the understanding of the vegetative incompatibility system. Consequently, this may improve hypovirulence biological control in North America where there is high vegetative incompatibility among the fungus strains (Milgroom and Cortesi, 2004). Success with hypovirulence may help keep the surviving American chestnuts alive while breeding for blight resistance is still ongoing.

It is also important to start making more efforts toward the understanding of gall wasp. Sequencing the genome of Ozark chinquapin (*C. ozarkensis*) will be an important tool since this species of chestnuts is resistant to the gall wasp (Anagnostakis, 2001).

The response of plants to abiotic stress is complex, and its complexity rises in tree species with long life cycles as chestnuts. In the case of *C. sativa*, there is a lack of systematic research on expressed genes and proteins, and involved metabolites and microbiome, allied to morphological and physiological responses to abiotic stresses. More studies in these subjects would permit directional genetic modification strategies for a more expedited species improvement besides breeding strategies (Figure 1), to cope with the rapidly advancing global climate change.

The chestnut species with more reports on abiotic stresses is *C. sativa*, most of them related to drought. Advances have been made in QTL identification and MAS has been used, however, a small number of genes involved in drought tolerance have been identified. The molecular markers used, reflect a limited part of the genome for such a complex stress. GBS of large sets of individuals was already used in *Castanea* species. This cost-effective technique could potentially improve the estimation of genetic diversity based on hundreds to thousands of genetic markers (Müller and Gailing, 2019).

Ultimately, global climate change will differently affect the ecosystems in which chestnut stands. Chestnut may adapt *via* alterations of physical traits, or it will occupy other ecosystems in new favorable geographical locations. The latter may be the most probable hypothesis (Pérez-Girón et al., 2020), which may guide the establishment of new chestnut orchards. The established regions of chestnut provenance are at risk of being seriously affected or even eliminated with climate change (Pérez-Girón et al., 2020). Their resilience and adaptation will depend on the extension of the climatic variations.

The contributions gathered for the past 40 years have given chestnut researchers great tools to support restoration and programs for developing sustainable control measures of biotic and abiotic stresses. This research also represents valuable knowledge that may be applied to other forest species, mainly related members of the Fagaceae family.

## Author contributions

PF wrote the introduction. PF and MC compiled and wrote the information regarding the biotic stresses. SS compiled and wrote the information regarding the abiotic stresses. RC compiled the work done on Dryocosmus kuriphilus. All authors reviewed the successive drafts of the manuscript, contributed to the article, and approved the submitted version.

## Footnotes

1
https://www.uniprot.org/uniprotkb/Q84QC2/entry
